# Evaluation of the effectiveness of transcranial direct current stimulation in reducing upper limb spasticity in patients after stroke: a systematic review of randomized controlled trials

**DOI:** 10.3389/fneur.2026.1879770

**Published:** 2026-08-26

**Authors:** Justyna Brożonowicz, Natalia Wołoszyn, Justyna Leszczak, Krzysztof Bylicki, Gabriela Ciąpała, Agnieszka Wiśniowska-Szurlej

**Affiliations:** 1 Faculty of Health Sciences and Psychology, Collegium Medicum, University of Rzeszów, Rzeszów, Poland; 2 Donum Corde Rehabilitation and Medical Care Center, Budy Głogowskie, Poland

**Keywords:** muscle spasticity, neurological rehabilitation, stroke, transcranial direct current stimulation, upper limb

## Abstract

**Introduction:**

Spasticity is a common motor disorder that develops after stroke, negatively impacting motor control and functional capacity. Non-pharmacological methods, including transcranial direct current stimulation (tDCS), are increasingly used in its treatment; however, the optimal stimulation protocol remains unclear. Therefore, the aim of this study was to conduct a systematic review of the literature regarding the efficacy of tDCS in treating upper limb spasticity in post-stroke patients.

**Methods:**

A systematic review of randomized controlled trials was conducted in accordance with the PRISMA guidelines. PubMed, Scopus, PEDro, Cochrane CENTRAL, ScienceDirect, Web of Science, and Google Scholar were searched using the following keywords and their combinations: transcranial direct current stimulation, stroke, muscle spasticity and upper limb. The eligibility criteria were defined according to the PICOTS framework. The risk of bias in the included studies was assessed using the Cochrane Risk of Bias 2 tool. The review was registered in PROSPERO (CRD420261363944).

**Results:**

Of the 2,797 records identified, nine publications met the eligibility criteria and were included in the analysis. These studies differed in terms of participant characteristics. In most cases, the intervention involved anodal stimulation of the primary motor cortex; however, the treatment parameters, including stimulation duration, current intensity, and number of sessions, were heterogeneous. A clear beneficial effect of tDCS on upper limb spasticity in patients after stroke was observed in three articles.

**Discussion:**

The available evidence regarding the effectiveness of tDCS in reducing upper limb spasticity after stroke remains inconclusive. Further high-quality studies using standardized research protocols are required.

**Systematic review registration:**

https://www.crd.york.ac.uk/PROSPERO/view/CRD420261363944, PROSPERO; registration number CRD420261363944.

## 1 Introduction

Spasticity is a disorder of sensorimotor control resulting from an upper motor neuron damage and manifesting as intermittent or sustained involuntary muscle activation. It is a motor dysfunction occurring in individuals with neurological conditions (1).

The exact prevalence of spasticity remains uncertain. It is estimated to affect approximately 38% of stroke survivors 12 months after stroke, 60%–90% of individuals with multiple sclerosis, 92% of individuals with primary lateral sclerosis (PLS), and approximately one in six individuals with traumatic brain injury. Spasticity also occurs in cerebral palsy and spinal cord injury (2).

Spasticity is a major challenge in the rehabilitation of individuals with neurological disorders, particularly those who have experienced a stroke (3). It is associated with impaired muscle strength and movement coordination, limited range of motion, and reduced manual dexterity. These conditions may limit mobility, transfers, and activities of daily living, thereby adversely affecting quality of life (2, 4).

In addition to pharmacotherapy, various non-pharmacological methods are used to treat spasticity (2). These include stretching exercises combined with other therapeutic modalities (5), extracorporeal shock wave therapy (ESWT) (6), vibration therapy (7), appropriately selected orthopedic equipment (8), repetitive transcranial magnetic stimulation (TMS) (9), transcutaneous electrical nerve stimulation (TENS) (10), and other rehabilitation interventions.

The use of tDCS to reduce spasticity has been increasingly investigated. Previous evidence suggests that stimulation lasting more than 20 min may be particularly effective in individuals younger than 60 years (11). However, Alashram et al., in a systematic review of randomized controlled trials (RCT), concluded that the evidence regarding the effects of tDCS on upper limb spasticity after stroke was limited and the optimal treatment dose remained unclear (12). Van Hoornweder et al. reported that tDCS-related improvements in upper limb function were more pronounced in individuals in the chronic phase after stroke (13).

Previous systematic reviews examining the use of tDCS to reduce upper limb spasticity in individuals after stroke included studies published up to 2021. Their conclusions generally emphasized the need for further investigation because of the limited and inconclusive evidence regarding the effectiveness of tDCS and the low methodological quality of many available studies (11, 12). The growing interest in the clinical applications of tDCS, the increasing number of publications in recent years, and the lack of a systematic review incorporating the most recent evidence, particularly studies published during the last 5 years of rapidly developing research on non-invasive brain stimulation, provided the rationale for conducting presented research. Therefore, the aim of this systematic review was to assess the effectiveness of tDCS in reducing upper limb spasticity after stroke.

## 2 Methods

### 2.1 Study design and registration

This systematic review was conducted in accordance with the Preferred Reporting Items for Systematic Reviews and Meta-Analyses 2020 statement (14). The review protocol was prospectively registered in the International Prospective Register of Systematic Reviews (PROSPERO; registration number CRD420261363944; available at: https://www.crd.york.ac.uk/PROSPERO/view/CRD420261363944).

### 2.2 Eligibility criteria

The review included studies published in Polish or English between January 1, 2015 and July 12, 2026. The eligibility criteria were defined according to the PICOTS framework:

(P) Population: Adults aged 18 years or older who had experienced an ischemic or hemorrhagic stroke and with spasticity in the affected upper limb;

(I) Intervention: tDCS used to reduce upper limb spasticity, either as a stand-alone intervention or in combination with other therapeutic modalities;

(C) Comparison: A control group receiving no intervention, a delayed intervention, or sham tDCS;

(O) Outcomes: Upper limb spasticity assessed before and after the intervention, with at least two measurements, using the Modified Ashworth Scale (MAS). The MAS is a 5-point scale ranging from 0 to 4, with higher scores indicating greater muscle tone (15);

(T) Timing: Immediate or short-term effects assessed after the intervention, as well as long-term effects assessed during follow-up;

(S) Study design: RCT conducted in accordance with the CONSORT guidelines.

Studies were excluded if upper limb spasticity resulted from central nervous system damage other than stroke, if tDCS was not used as an intervention aimed at reducing spasticity, or if the study design was other than a RCT (meta-analyses, literature reviews, cross-sectional studies, longitudinal studies).

### 2.3 Search strategy

The following databases and sources were searched: PubMed, Scopus, PEDro, Cochrane CENTRAL, ScienceDirect, Web of Science, and Google Scholar. The search used the following MeSH terms, where applicable: transcranial direct current stimulation, stroke, muscle spasticity, and upper extremity. The following entry terms and additional keywords were also used: tDCS, cerebral stroke, cerebrovascular accident, spastic, spasticity, upper limb, arm, and hand. The terms were used in various database-specific combinations. Detailed search strategies for each database, including the search period, date of the final search, keywords and applied search parameters are provided in Supplementary material S1.

### 2.4 Study selection

The study selection process was conducted independently by two authors (JB and NW). The authors assessed the eligibility of the retrieved records according to the predefined PICOTS criteria, initially on the basis of titles and abstracts. When the information required to determine eligibility was insufficient, the full-text article was reviewed. After the searches had been completed, the results were compared and duplicates were removed. Any disagreements regarding study eligibility were resolved through discussion with a third author (JL).

### 2.5 Data collection and extraction process

Data from the selected studies were extracted into a standardized Microsoft Excel spreadsheet (Office LTSC 2024). Data extraction was performed independently by two researchers (JB and NW) and supervised by a third author (JL). The following information was extracted from the included studies:

(1) Study design.
(2) Characteristics of the study group, including: (a) population size, (b) mean age, (c) type of stroke, (d) time since stroke onset, (e) side of paresis, (f) level of spasticity before the intervention.
(3) Description of the intervention in the study group: (a) type of tDCS stimulation, (b) duration, (c) current intensity during the procedure, (d) electrode location, (e) number of treatment sessions, (f) any additional interventions, (g) occurrence of adverse effects; (h) completion of the intervention for the entire group.
(4) Description of the intervention in the sham and/or control group.
(5) Obtained results – including the level of spasticity according to the MAS scale.

### 2.6 Risk of bias

The risk of bias in the included studies was assessed using the Revised Cochrane Risk of Bias Tool for Randomized Trials (RoB 2). Five domains were evaluated: bias arising from the randomization process, bias due to deviations from the intended interventions, bias due to missing outcome data, bias in measurement of the outcome, and bias in selection of the reported result. An overall risk-of-bias judgement was also assigned to each study. The risk of bias was classified as low, high, or presenting some concerns (15, 16).

## 3 Results

### 3.1 Study selection

**Figure 1:**
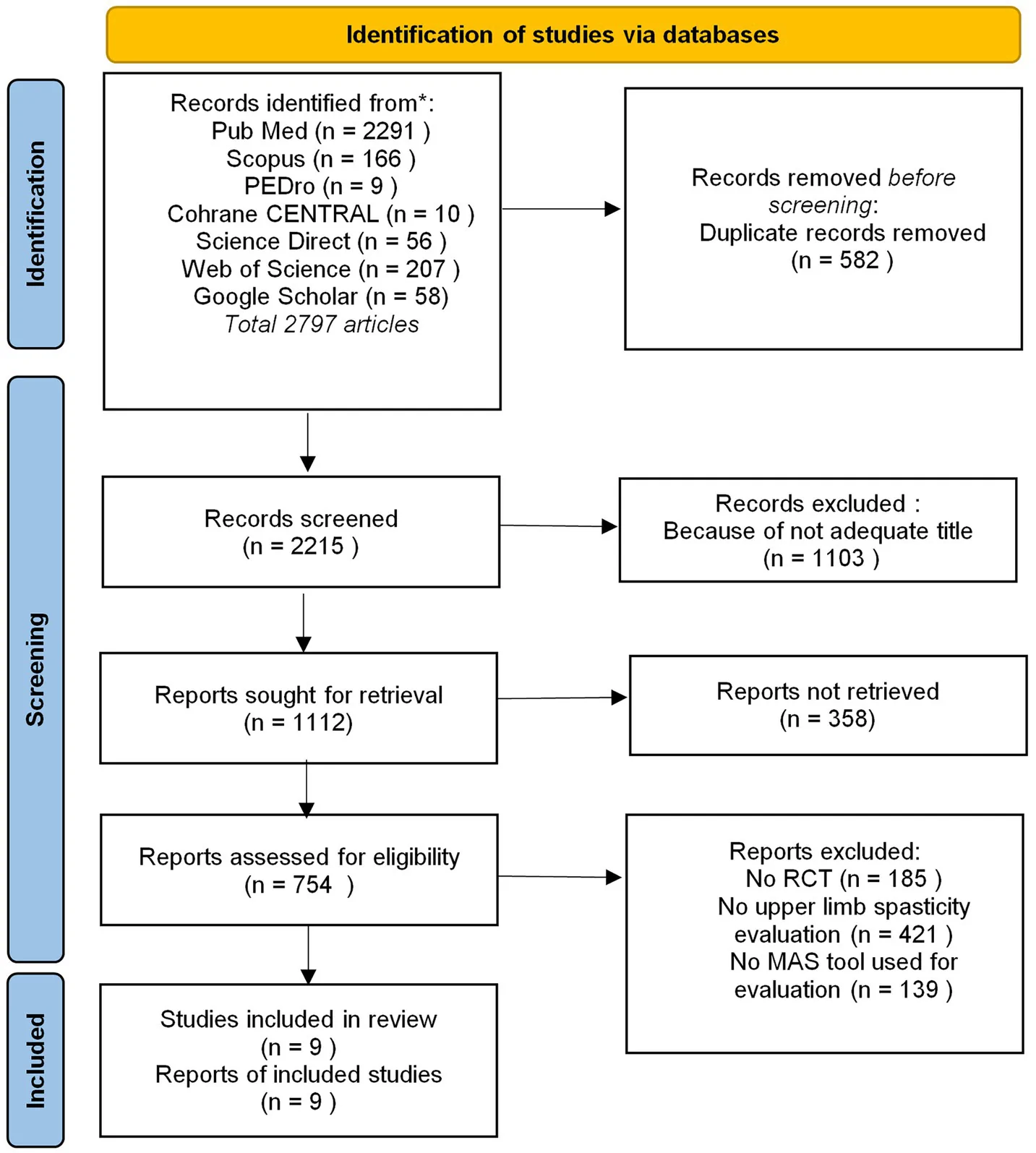
PRISMA 2020 flow diagram (14). Figure prepared based on original data collected by the authors.

The search identified a total of 2,797 records. After duplicates had been removed, 2,215 reports remained for further screening. Eligibility was assessed sequentially on the basis of titles, abstracts, and full texts. Of the 754 full-text reports assessed, 745 did not meet the eligibility criteria. Ultimately, nine RCT were included in the qualitative synthesis and risk-of-bias assessment. The study selection process is presented in the PRISMA flow diagram (Figure 1).

### 3.2 Risk of bias

**Figure 2:**
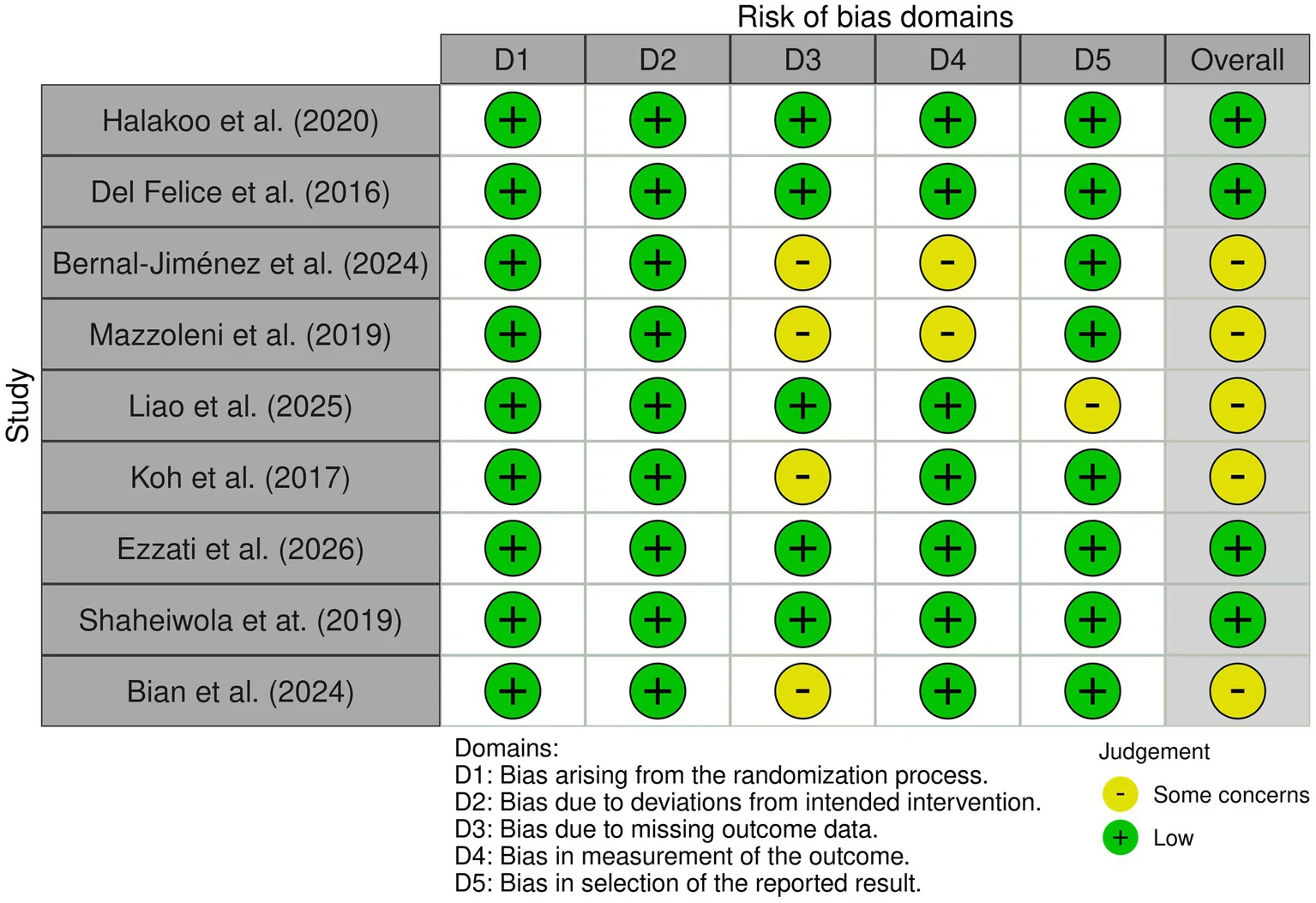
Robvis: quality risk-of-bias assessment figure (39).

Of the nine studies included in the review, four were assessed as having a low overall risk of bias (17, 18, 19, 20), whereas five were judged as presenting some concerns (21, 22, 23, 24, 25). Concerns were most frequently identified in domain 3, in relation to missing outcome data (21, 23, 24, 25), and domain 4, relating outcome measurement (21, 23) (Figure 2).

### 3.3 Characteristics of the studies

#### 3.3.1 Type and time since stroke

In most of the included studies, the surveyed population comprised individuals who had experienced either an ischemic or hemorrhagic stroke. However, patients with ischemic stroke constituted the majority of participants in each of these studies (17, 18, 19, 20, 21, 24, 25, 26). The mean age of participants ranged from 56.1 to 68.7 years. The studies differed substantially in the time elapsed since stroke onset. The inclusion criteria ranged from the immediate post-stroke onset (23) to the chronic stage (26). In most studies, participants had a baseline upper limb spasticity score of at least 1 and no more than 3 on the MAS.

#### 3.3.2 tDCS intervention – electrode location, duration of a single treatment and the entire intervention

Seven studies used anodal tDCS (18, 19, 20, 21, 23, 24, 26), whereas two studies used cathodal stimulation (17, 25). In most studies using anodal stimulation, the anodal electrode was placed over the primary motor cortex (M1) of the affected hemisphere, and the cathodal electrode was placed over the contralateral supraorbital region (18, 23, 24, 26). In one study, the anodal electrode was placed over the ipsilesional M1, whereas the cathodal electrode was placed over the contralateral M1 (21). In the study using cathodal stimulation, the cathodal electrode was placed over the M1 contralateral to the lesion, and the anodal electrode was placed over the contralateral supraorbital region (17). Two studies used multichannel stimulation protocols (19, 25).

In most studies, a single stimulation session lasted 20 min (17, 18, 21, 23, 26), whereas one study used 30-min sessions (24). The current intensity was 1 mA in two studies (17, 19), 1.5 mA in two studies (24, 25), and 2 mA in five studies (18, 20, 21, 23, 26). The total number of sessions varied considerably, ranging from 5 to 30. Interventions were administered daily in two studies (21, 23) and every other day in another two (24, 26). Meanwhile, two publications failed to report the frequency of stimulation (17, 18).

Most reports indicated that tDCS was not associated with adverse effects or substantial discomfort (20, 21, 23, 24, 25). Two studies reported transient burning, itching, or tingling beneath the electrodes, primarily at the beginning of the stimulation session (18, 22). Another two did not provide information regarding adverse effects (17, 19). In four trials, all participants who had been recruited and randomized completed the planned intervention (17, 19, 20, 25). In contrast, five publications reported participant attrition during the intervention period (18, 21, 22, 23, 24). The reported reasons for attrition included health-related problems, acute medical events, hospitalization, personal circumstances precluding on-site participation, and concerns regarding infection during the COVID-19 pandemic.

#### 3.3.3 tDCS placebo stimulation

All included studies used sham tDCS in the sham group. In most protocols, electrode placement and the overall duration of the procedure were identical to those used in the active-stimulation group. However, the current was delivered only during the initial phase to produce sensations similar to those experienced during active stimulation and was subsequently discontinued (17, 18, 19, 20, 23, 24, 25, 26). In one study, direct current stimulation was replaced by alternating current stimulation after the initial activation period (21).

#### 3.3.4 Additional interventions implemented in conjunction with tDCS

In seven publications, tDCS was combined with an additional rehabilitation intervention (18, 20, 21, 23, 24, 25, 26), whereas in two studies it was used as the sole treatment (17, 19). These concomitant modalities included upper limb robot-assisted therapy (21, 23) and functional electrical stimulation of the wrist extensor muscles (18, 20). Mirror therapy combined with functional exercises was investigated in one article (26), while another focused on passive exercises performed under cutaneous anesthesia alongside standard physiotherapy (24). Finally, routine rehabilitation was implemented in two studies (24, 25).

#### 3.3.5 Impact of the intervention on the MAS score

Based on post-intervention MAS scores, a clear beneficial effect of tDCS on upper limb spasticity was observed in three studies (17, 18, 26). These findings included reductions in muscle tone in the flexor carpi radialis, finger flexors, and elbow flexors and extensors. One study reported a statistically significant reduction in spasticity; however, the effect was short-lived and not maintained at the 3-month follow-up (24). In another report, reductions in spasticity were documented in both the active-intervention and sham-tDCS groups. This finding was considered inconclusive because the observed improvement may have resulted from the concomitant intervention rather than tDCS itself (20). Four studies reported no statistically significant reduction in spasticity (19, 21, 23, 25) (Table 1).

**Table 1:** Detailed characteristics of the studies included in the analysis.

Study characteristics	Author and year of publication								
	Halakoo et al. (2021) (18)	Del Felice et al. (2016) (17)	Bernal-Jiménez et al. (2024) (21)	Mazzoleni et al. (2019) (23)	Liao et al. (2025) (26)	Koh et al. (2017) (24)	Ezzati et al. (2026) (19)	Shaheiwola et al. (2019) (20)	Bian et al. (2024) (25)
Research design	Double-blind RCT	Double-blind, crossover RCT	Prospective double-blind RCT	Prospective single-blind RCT	Prospective randomized double-blind RCT	Prospective randomized double-blind RCT	Double-blind RCT	Double-blind RCT	Double-blind RCT
Characteristics of the study group									
Population size	35	10	19	39	36	25	38	30	60
Mean age in the study group	62.61	62.00	59.50	68.74	59.10	56.10	64.34	50.60	61.47
Stroke type (I/H)	32/0	10/0	12/7	NI	19/17	18/7	38/0	NI	51/9
Mean time since stroke onset [months]	3–6	>9 (mean 27,6)	16.8	≤1	42.75	11.9	8.5	17	<1
Side of paresis (L/R)	NI	NI	8/11	17/22	18/18	14/11	NI	14/16	39/21
Spasticity level before intervention (MAS)	>1	≥1	0–4	1–2	< 3	< 3	≥1	Mean 5,32	1–3
Description of the tDCS intervention in the study group									
Type of tDCS stimulation	Anodal	Cathodal	Anodal	Anodal	Anodal	Anodal	Anodal	Anodal	Cathodal
Duration of a single session [minutes]	20	20	20	20	20	30	20	20	20
Current intensity during the treatment [mA]	2	1	2	2	2	1.5	1	2	1.5
Electrode location (anode)	M1 field of the affected hemisphere	The suprafrontal region on the opposite side; in the case of double tDCS stimulation over M1	Over the ipsilateral M1	M1 of the affected hemisphere	M1 or premotor cortex PMC (depending on the group)	Over the M1 on the side of the lesion	(M1, C3/C4) and (DLPFC, F3/F4) in the affected cerebral hemisphere	Over the abductor pollicis brevis muscle hot spot on the lesioned hemisphere	4 return electrodes 3.5 cm away from the central electrode
Electrode location (cathode)	Suprafrontal region on the opposite side	M1 of the uninvolved hemisphere corresponding to the cortical representation of the interosseous muscles of the hand; in the case of double tDCS over M1 on the side opposite the lesion.	Contralateral M1	Above the orbit on the side opposite the lesion	Contralateral orbitofrontal cortex	Over the Mi contralateral to the lesion	Above the supraorbital region on the side opposite the lesion	On the contralateral symmetrical area of non-lesioned hemisphere.	ipsilesional M1
Number of treatment sessions:	10	5	20	30	20	24	5	20	15
Device used	NI	TransQE, IOMED, Salt Lake City, UT	DC-Stimulator	HDC kit (Newronika Srl, Milano, Italy)	StarStim, Neuroelectrics, Barcelona, Spain	Intelect®125 Mobile Combo (Chattanooga, USA)	NI	BrainStim stimulator, E.M.S.s.r.l., Italy	HD-tDCS adaptor (Soterix Medical, New York, NY, USA)
Additional interventions in the treatment protocol:	FES of the extensor carpi radialis muscles	None	Robotic therapy of the upper limb using the AMEDO robot during tDCS stimulation.	Robotic wrist training based on reaching tasks using the InMotion WRIST robot	Mirror therapy and functional exercises (total 90 min per day)	Bilateral cutaneous anesthesia and passive movements of the affected hand, regular rehabilitation programs occupational therapy and physiotherapy, speech therapy	None	FES therapy with task-oriented movements during 1 h	iTBS, 3 weeks routine rehabilitation treatment
Adverse effects	Itching and tingling	NI	None	None	Tingling and itchy but only at the beginning of each session	None	NI	None	None
Intervention in the sham group	Sham a-tDCS and FES	All All participants underwent a week of sham tDCS stimulation 4 weeks before the first experimental block.	Sham-tCDS	Sham-tDCS	Sham-tCDS	Sham bilateral tDCS	5 sessions of active anodal tDCS targeting M1 and sham stimulation targeting DLPFC [a-tDCS M1-DLPFC].	Sham tDCS+FES therapy	II group sham tDCS + iTBS III group: tDCS + sham iTBS
Intervention in the control group	FES and standard physiotherapy	No control group	No control group	No control group	No control group	No control group	No control group	No control group	No control group
Completion of the intervention for the entire group	No	Yes	No	No	No	No	Yes	Yes	Yes
Results obtained	Reduction in MAS (*p* < 0.001) of the flexor carpi radialis muscle at rest was demonstrated, immediately and 1 month after the intervention in the intervention group (M1 a-tDCS), compared to the control group and the sham group	Both tDCS protocols demonstrated a reduction in spasticity scores on the MAS scale for the finger flexors, wrist flexors, and elbow flexors and extensors (Week 1 > Week 2: *p* = 0.008) in all subjects. Cathodal tDCS was slightly more effective than dual tDCS in reducing spasticity of the distal upper limb in patients with chronic stroke.	No reduction in spasticity was observed in any group, either immediately after the intervention or after the 3-month follow-up period.	No statistically significant differences were observed in mean MAS values after the intervention in both groups, as well as in the between-group comparisons.	A statistically significant reduction in spasticity was observed in the PMC group (in which tDCS stimulation was applied to the premotor cortex) (*p* = 0.01).	The real tDCS group showed a trend toward a small immediate effect within 3 days of completing the intervention (ηp2 = 0.02–0.04) in reducing wrist and finger flexor spasticity. No difference in treatment effect between the two groups at the 3-month follow-up.	No significant changes were observed in either group after the intervention for elbow or wrist spasticity. Comparisons between groups after the intervention also revealed no significant differences in elbow (*p* = 0.418) or wrist (*p* = 0.325) spasticity.	MAS results indicated that both protocols were able to induce an overall reduction in upper limb spasticity. However, there was no significant difference between the two protocols in terms of spasticity reduction.	There were no statistically significant differences in the level of spasticity of the pectoralis major and biceps brachii muscles before and after the intervention between the 3 groups.

I/H - ischemic/hemorrhagic stroke; L/R - left/right; NI - no information; MAS - Modified Ashworth Scale; FES - functional electrical stimulation; M1 - primary motor cortex; RCT - randomized controlled trial; PMC - premotor cortex; DLPFC - dorsolateral prefrontal cortex; HD-tDCS - high-definition transcranial direct current stimulation; iTBS - intermittent theta burst stimulation.

Because of substantial clinical and methodological heterogeneity, a meta-analysis was not considered appropriate. A structured qualitative synthesis was therefore conducted. The most frequently used intervention characteristics were a current intensity of 2 mA, a session duration of 20 min, treatment during the chronic phase after stroke, and at least 10 stimulation sessions. However, no consistent relationship between individual intervention characteristics and treatment effectiveness could be established because of considerable differences in electrode montage, number of sessions, and concomitant rehabilitation interventions (Table 2). Table 3 presents an effect-direction synthesis of the included studies based on MAS scores.

**Table 2:** Synthesis of tDCS intervention characteristics and factors associated with clinical outcomes.

Intervention parameter	Predominant characteristics of the applied tDCS protocols	Studies, (R/A)	Studies reporting positive outcomes (P/R)
Stroke phase	Chronic	6/9	3/6
tDCS intensity	2 mA	5/9	3/5
Session duration	20 min	8/9	4/8
Number of sessions	≥10 sessions	7/9	3/7
Electrode montage	M1–supraorbital	4/9	2/4
Type of stimulation	Anodal tDCS	7/9	3/7
Additional interventions		7/9	
	FES	2/9	2/2
	Robot-assisted therapy	2/9	0/2
	Conventional rehabilitation	2/9	0/2
	iTBS combined with conventional rehabilitation	1/9	0/1

tDCS, transcranial direct current stimulation; mA, milliampere; min, minutes; M1, primary motor cortex; supraorbital, contralateral supraorbital region; FES, functional electrical stimulation; iTBS, intermittent theta burst stimulation. R/A indicates the number of studies reporting a given intervention characteristic (R) divided by the total number of included studies (A). P/R indicates the number of studies reporting a positive outcome (P) divided by the number of studies reporting the corresponding intervention characteristic (R). A positive outcome was defined as a statistically significant reduction in upper limb spasticity following the tDCS intervention.

**Table 3:** Effect-direction synthesis of the included studies based on MAS scores.

Study	Outcome	Direction of effect
Halakoo et al. (2021) (18)	Level of spasticity in MAS	↑
Del Felice et al. (2016) (17)		↑
Bernal-Jiménez et al. (2024) (21)		↓
Mazzoleni et al. (2019) (23)		↑
Liao et al. (2025) (26)		↔
Koh et al. (2017) (24)		↓
Ezzati et al. (2026) (19)		↓
Shaheiwola et at. (2019) (20)		↔
Bian et al. (2024) (25)		↓

↑ – positive effect.

↓ – negative effect.

↔ – no effect/mixed effect/conflicting findings.

## 4 Discussion

The aim of this systematic review was to assess the effectiveness of tDCS in reducing upper limb spasticity after stroke. The analysis of nine RCT did not demonstrate a consistent therapeutic effect, indicating that the effects of tDCS on muscle tone remain difficult to predict. These findings reflect the complex pathophysiology of spasticity and the multifactorial nature of the response to neuromodulation (2).

### 4.1 Mechanism of action

The effects of tDCS are associated with changes in cortical excitability and modulation of neuroplasticity. Anodal stimulation generally increases cortical excitability, whereas cathodal stimulation decreases it. These effects may contribute to the partial restoration of the interhemispheric balance disrupted after stroke (9). Moreover, tDCS may indirectly influence subcortical structures and the spinal cord by modulating interneuronal activity and stretch reflexes (27). These mechanisms may be relevant to the pathophysiology of spasticity, which is associated with impaired regulation of segmental reflexes and excessive reflex-arc activity (28).

### 4.2 Interpretation of the results in light of the current literature

The findings suggest that tDCS may reduce spasticity as assessed using the MAS; however, the effects are inconsistent and appear to depend on the intervention conditions. Three studies reported significant improvements (17, 18, 26), whereas four did not identify significant changes (20, 21, 23, 25). In one trial, the observed effects were short-lived (24). Ezzati et al. (19) reported an overall reduction in upper limb spasticity following both stimulation protocols; however, no significant differences were observed between the protocols. These findings allign with current evidence indicating the limited effectiveness of tDCS in spasticity reduction. A meta-analysis by Huang et al. demonstrated a moderate reduction in spasticity, particularly in individuals younger than 60 years. The effects also depended on the stimulation parameters and type of stroke (11). Similar findings were reported by Alashram et al., who concluded the therapeutic effects as insufficient to support clear clinical recommendations (12). In contrast, Elsner et al. found no significant effect of tDCS on spasticity and emphasized the lack of studies evaluating long-term outcomes (29). The available evidence suggests that the effectiveness of tDCS may depend on the stimulation protocol (22), dose (30), and individual patient characteristics (31). This may partly explain the inconsistent results and indicates that therapeutic protocols have not yet been sufficiently optimized.

### 4.3 Inconsistency of results

The findings of the presented review are consistent with previous reports of variable therapeutic effects of tDCS. Meta-analyses have shown that multisession stimulation may improve upper limb motor function, with more pronounced effects observed during the chronic rather than acute phase after stroke (13, 30, 32). However, the absence of standardized protocols and the small sample sizes used in individual studies substantially complicate the interpretation of the findings (31, 33). The available evidences suggest that tDCS may exert a greater effect on motor function than on spasticity (34). This observation is supported by Ezzati et al., who reported overall reductions in MAS scores but no differences between the stimulation protocols (19). Similarly, Shaheiwola et al. and Bian et al. found no significant between-group differences in muscle tone despite improvements in selected functional outcomes (20, 25). Abdullahi et al. reported that tDCS improved upper limb function after stroke, whereas its effects on muscle tone remained limited (33). Comparable findings were reported by Van Hoornweder et al., who observed greater effects on motor function than on muscle tone (13). These findings suggest that the primary effects of tDCS may relate to motor control rather than to a direct reduction in spasticity. Spasticity is a multifactorial phenomenon involving both neurophysiological and biomechanical components (4). Therefore, interventions acting primarily at the cortical level may not address all mechanisms contributing to increased muscle tone.

### 4.4 Possible reasons for the discrepancy in results

One factor explaining the inconsistent findings is the substantial heterogeneity of the study populations. The time since stroke onset also differed considerably among studies and may directly influence the response to treatment. As noted by Li et al., the characteristics of spasticity change over time. Neurological components predominate during the acute phase, whereas structural changes in muscles and soft tissues become more important during the chronic phase, potentially affecting the reaction to neuromodulation (4).

Stimulation parameters may also impact the therapeutic outcomes. Huang et al. demonstrated that the effects of tDCS depended on the dose, number of sessions, and electrode placement (11). The absence of standardized intervention protocols limits comparisons between studies and makes it difficult to identify optimal therapeutic regimens.

The side of stimulation may also be important. Most studies have focused on the primary motor cortex. However, the findings of Liao et al. suggest that stimulation of the premotor cortex may be more effective for selected functional outcomes (26), possibly because of the involvement of this region in movement planning and sensorimotor integration. Further research is required to identify the optimal stimulation target.

### 4.5 The importance of combination therapy

In most of the included reports, tDCS was combined with other rehabilitation interventions. Halakoo et al. reported significant improvements in clinical outcomes when tDCS was supplemented with functional electrical stimulation (18). In studies integrating tDCS with robot-assisted therapy, the effects were less pronounced (21, 23). Shaheiwola et al. also conjoined tDCS with functional electrical stimulation; however, no significant improvement in MAS scores was observed (20). Similarly, Bian et al. coupled tDCS with iTBS and routine rehabilitation, but the protocol did not produce a significant reduction in spasticity (25). These findings indicate that the effects of tDCS may vary depending on the type of concomitant rehabilitation intervention.

### 4.6 The importance of methodological quality and risk of bias in the interpretation of results

The risk-of-bias assessment using the RoB 2 tool showed that four studies (17, 18, 19, 20) had a low overall risk of bias, whereas five reports (21, 23, 24, 25, 26) were judged as presenting some concerns. None of the studies was assessed as having a high risk of bias. Similar limitations in the quality of the evidence have been reported in recent systematic reviews, particularly small sample sizes, heterogeneous study populations, and variation in stimulation parameters (35, 36).

Beneficial therapeutic effects were observed in studies with a low risk of bias (17, 18), whereas the publication by Mazzoleni et al. (23), which reported a positive outcome, was classified as presenting some concerns. These observations are consistent with the meta-analysis by Huang et al. (11), which demonstrated beneficial effects of anodal tDCS while also emphasizing the low quality of the available evidence. Wang et al. reached similar conclusions, reporting potentially beneficial effects of non-invasive brain stimulation while emphasizing the need for further studies of high methodological quality (36).

The most frequently identified concerns related to missing outcome data and outcome measurement, whereas the randomization process was generally assessed as having a low risk of bias. Similar objections were reported by Alashram et al. (12). Although the overall methodological quality of the available studies was relatively good, the limited number of publications and their considerable heterogeneity prevent definitive identification of the optimal tDCS parameters or electrode configurations.

### 4.7 Clinical significance

From a clinical perspective, tDCS is a potentially useful adjunct to post-stroke rehabilitation. Its main practical advantages include its non-invasive nature, good tolerability, favorable safety profile, and relatively low cost compared with TMS. Its ease of application and the compact size of tDCS devices may facilitate implementation in both inpatient and outpatient rehabilitation environment. In addition, stimulation can be administered concurrently with functional therapy. However, its routine utilization requires appropriately trained personnel, standardized procedures, and adherence to established safety recommendations (37). The Third Stroke Recovery and Rehabilitation Roundtable also emphasized the need to standardize research methodology and develop consistent therapeutic protocols (38). An important advantage of tDCS is the potential to integrate this method with conventional rehabilitation and adjunctive therapies, including functional electrical stimulation (18), robot-assisted therapy (21, 23), and other neuromodulation techniques (25). Current recommendations suggest that tDCS may have greater clinical potential when used as a component of combined therapy rather than as a stand-alone treatment for post-stroke spasticity (38).

Despite the growing number of studies, clear patient-selection criteria and optimal stimulation parameters have not yet been established. The available evidence indicates that treatment should be individualized according to the phase after stroke, severity of impairment, and rehabilitation goals (29). At the same time, the limited number of reports specifically addressing spasticity, the heterogeneity of the available evidence, and the lack of standardized protocols indicate that tDCS should currently be regarded as a promising adjunct rather than a standard component of clinical management. Its widespread implementation requires further well-designed, multicenter clinical trials using standardized protocols (38).

### 4.8 Study limitations

The included studies had several limitations. The small sample sizes limit the generalizability of the findings. In addition, substantial methodological heterogeneity was observed because of differences in stimulation protocols, intervention duration, and participant characteristics. The review included only studies using the MAS to assess spasticity. This decision was intended to maximize the consistency of the methodological assumptions and improve the clarity of the analysis. However, this criterion may have resulted in the exclusion of potentially relevant studies assessing spasticity using other measurement instruments. All included studies used the MAS, a standardized clinician-rated scale widely used in clinical research. However, MAS assessments may be influenced by the examiner’s experience and other examiner-related factors. This potential source of measurement variability should be considered when interpreting the findings. Furthermore, the review was based on a qualitative synthesis without a meta-analysis, which limits the ability to quantitatively estimate the therapeutic effects. Another limitation of this review was the inclusion of only studies published in Polish and English. This language restriction may have had some impact on the scope of the evidence included in the analysis.

## 5 Conclusion

The available evidence from RCT does not provide conclusive support for the effectiveness of tDCS in reducing upper limb spasticity after stroke. Considerable clinical heterogeneity among participants, variation in intervention protocols, small sample sizes, and the absence of a quantitative synthesis limit confidence in the findings. Although some studies reported beneficial effects, the overall evidence remains inconsistent and does not currently support firm clinical recommendations. Further well-designed studies are required to develop standardized tDCS-based therapeutic protocols and to identify the groups of patients after stroke who may be most likely to benefit from this intervention.

## Data Availability

The data analyzed in this study is subject to the following licenses/restrictions: the database can be made available at the editor or reader’s request. Requests to access these datasets should be directed to jbrozonowicz@ur.edu.pl.
